# Mitochondrial Proteins (e.g., VDAC, Bcl-2, HK, ANT) as Major Control Points in Oncology

**DOI:** 10.3389/fonc.2014.00365

**Published:** 2014-12-15

**Authors:** Catherine Brenner, Antoinette Lemoine

**Affiliations:** ^1^INSERM UMR-S 769, LabEx LERMIT, Châtenay-Malabry, France; ^2^IFR141 – IPSIT, CIBLOT Platform, Châtenay-Malabry, France; ^3^Université de Paris-Sud, Faculté de Pharmacie, Châtenay-Malabry, France; ^4^Université Paris-Sud, Institut André Lwoff, Villejuif, France

**Keywords:** mitochondria, apoptosis, oncogenes, bcl-2-associated X protein, metabolism

Mitochondria are fascinating organelles, at least in part, because two membranes with very different permeability properties and functions surround them. These membranes contribute to the intracellular compartmentation between mitochondria and the cytosol in eukaryotic cells. Thus, lipids (e.g., phospholipids, cholesterol, cardiolipin), membrane proteins [e.g., voltage-dependent anion channel (VDAC), ion exchangers, mitochondrial carriers such as adenine nucleotide translocase (ANT)], as well as soluble intermembrane space proteins cooperate to allow the channeling of metabolites (e.g., ATP, ADP), ions (e.g., calcium, potassium, sodium) and water across mitochondrial membranes. An increasing body of literature suggests that mitochondrial proteins behave also as control point in oncology (1, 2). For example, they can be involved (i) in the metabolic shift of cancer cells from oxidative phosphorylation to glycolysis via its binding to cytosolic proteins such as hexokinase II (i.e., Warburg effect), (ii) in the control of calcium fluxes between the endoplasmic reticulum (ER) and the mitochondrion via interaction with the inositol-3-phosphate receptor (IP3R), (iii) in the control of inner mitochondrial membrane potential (ΔΨm) via interaction with tubulin and finally, (iv) in the regulation of mitochondrial membrane permeability via interaction with Bax/Bcl-2 family members and/or the mitochondrial permeability transition pore complex (PTPC). Moreover, some proteins, via a shift in their expression, appear to be critical for the cancer cell response to chemotherapy (e.g., cisplatin, oxaliplatin, carboplatin). However, how these diverse functions are modulated and coordinated in cancer cells to control the balance between life and death is still largely unknown and requires extensive review and discussion to broaden our view of the role of mitochondrial proteins in oncology, the role of upstream signaling pathways controlling mitochondrial function and how these proteins and/or signaling pathways might be selective target for anti-cancer therapy.

Thus, this research topic presents seven manuscripts, which reviewed the current knowledge of mitochondrial proteins and polyprotein complexes as druggable target for anti-cancer therapy and also explored new putative targets. Thus, Suh et al. reviewed the literature on the anti-cancer potential of the modulation of the permeability transition pore, a megachannel in the inner membrane (3). Sutendra and Michelakis proposed to reverse mitochondrial suppression, which is an hallmark of many cancer cells, with metabolic-modulating drugs, like pyruvate dehydrogenase kinase (PDK) inhibitors or M2 isoenzyme of pyruvate kinase (PKM2) activators as a novel anti-cancer strategy (4). In a similar perspective, Rasola and Chiara discussed the mechanistic interactions among oncogenic kinase pathways, glycogen synthase kinase (GSK-3) activity and subsequent modulation of mitochondrial functions that shape the pro-survival phenotype of cancer cells, such as control of redox homeostasis and inhibition of the mitochondrial permeability transition pore (5). In addition, Shoshan-Barmatz et al. review current evidence pointing to the function of VDAC1 in cell life and death, and highlight these functions in relation to cancer (6). Alternatively, Fulda proposed to shift the balance of mitochondrial apoptosis to by pass evasion of apoptosis and treatment resistance in human cancers (7).

Ishii et al. reviewed the accumulating knowledge on pyrvinium pamoate, an FDA-approved anthelmintic, as an anti-cancer drug targeting mitochondrial respiration (8). Interestingly, this drug acts synergistically with dexamethasone to block the proliferation if human myeloma cells and might be used in combination.

In a more exploratory approach, Kang and Pervaiz explored the crosstalk between Bcl-2 family and Ras family small GTPases (9). They analyzed their work and the recent literature to show how this molecular crosstalk can regulate the cell fate. Previously, they showed a converging role of Rac1 and Bcl-2 to promote a pro-oxidant state, frequently observed in cancer cells. Despite the complexity of GTPases family, they argued that the modulation of the physical interactions between guanine exchange factors (GEF) and GTPases could open new avenues for future redox-based therapeutic designs.

We hope that this research topic provides new insights into the role of mitochondrial proteins as major control points in oncology and help the reader to elaborate new strategies for cancer therapy.

## Conflict of Interest Statement

The authors declare that the research was conducted in the absence of any commercial or financial relationships that could be construed as a potential conflict of interest.
